# Circulating angiopoietin-like protein 8 (betatrophin) association with HsCRP and metabolic syndrome

**DOI:** 10.1186/s12933-016-0346-0

**Published:** 2016-02-05

**Authors:** Mohamed Abu-Farha, Jehad Abubaker, Irina Al-Khairi, Preethi Cherian, Fiona Noronha, Sina Kavalakatt, Abdelkrim Khadir, Kazem Behbehani, Monira Alarouj, Abdullah Bennakhi, Naser Elkum

**Affiliations:** Biochemistry and Molecular Biology Unit, Dasman 15462, P.O. Box 1180, Kuwait City, Kuwait; Dasman Diabetes Institute, Kuwait City, Kuwait; Sidra Medical and Research Center, Clinical Epidemiology, P.O. Box 26999, Doha, Qatar

## Abstract

**Background:**

ANGPTL8 also called betatrophin is a regulator of lipid metabolism through its interaction with ANGPTL3. It has also been suggested to play a role in insulin resistance and beta-cell proliferation. Based on its function, we hypothesized that ANGPTL8 will play a role in Metabolic Syndrome (MetS). To test this hypothesis we designed this study to measure ANGPTL8 level in subjects with MetS as well as its association with high sensitivity C-reactive protein (HsCRP) level in humans.

**Methods:**

ANGPTL8 level was measured using ELISA in subjects with MetS as well as their controls, a total of 1735 subjects were enrolled. HsCRP was also measured and its association with ANGPTL8 was examined.

**Results:**

ANGPTL8 level was higher in subjects with MetS 1140.6 (171.9–11736.1) pg/mL compared to 710.5 (59.5–11597.2) pg/mL in the controls. Higher levels of ANGPTL8 were also observed with the sequential increase in the number of MetS components (*p* value = <0.0001). ANGPTL8 showed strong positive correlation with HsCRP (*r* = 0.15, *p* value = <0.0001). Stratifying the population into tertiles according to the level of HsCRP showed increased ANGPTL8 level at higher tertiles of HsCRP in the overall population (*p* value = <0.0001).A similar trend was also observed in MetS and non-MetS subjects as well as in non-obese and obese subjects. Finally, multiple logistic regression models adjusted for age, gender, ethnicity and HsCRP level showed that subjects in the highest tertiles of ANGPTL8 had higher odds of having MetS (odd ratio [OR] = 2.3, 95 % confidence interval [CI] = (1.6–3.1), *p* value <0.0001.

**Conclusion:**

In this study we showed that ANGPTL8 is increased in subjects with MetS and it was significantly associated with HsCRP levels in different subgroups highlighting its potential role in metabolic and inflammatory pathways.

## Background

MetS is a cluster of metabolic risk factors that are associated with an increased risk of metabolic related diseases such as cardiovascular diseases (CVD) and type 2 diabetes (T2D) [1–3]. In a meta-analysis study, MetS was shown to cause a twofold increase in cardiovascular outcomes and a 1.5-fold increase in all-cause mortality [1]. Central obesity, dyslipidemia, elevated blood pressure, elevated fasting glucose and insulin resistance are the most pivotal components of MetS [4–7]. MetS is also characterized by a chronic low grade inflammation state which can explain the increased CVD and T2D risk [8]. HsCRP is a proinflammatory protein that is produced by the liver in response to cytokine production due to acute inflammation. HsCRP is a marker of low grade inflammation which was shown in many studies to be higher in subjects with MetS. It was also shown that increased level of HsCRP is associated with increased risk of CVD and T2D [9–11]. Furthermore, it was shown that adding HsCRP to the definition of MetS will increase its predictive power of CVD and T2D [9–12].

ANGPTL8 is another liver produced protein that has been recently given increased attention due to its role in lipid metabolism and insulin resistance [13–15]. ANGPTL8 showed high level of expression in white, brown adipose tissues as well as the liver [14, 15]. It was suggested that ANGPTL8 is an atypical ANGTPL protein which closely interacts and regulates ANGTPL3 affecting levels of TG, HDL-C and LDL-C [13, 15–17]. ANGPTL8 or betatrophin- as called by Yi et al. has been suggested to increase beta-cell proliferation and beta-cell mass in insulin resistance mouse model [18]. However, recent data challenged the findings of Yi et al. and it was shown that ANGPTL8 does not play a role in beta-cell proliferation under insulin resistance conditions [19, 20].

We have recently showed that ANGPTL8 is increased in T2D and was associated with increased C-peptide level in non-diabetic subjects [21, 22]. However, the role of ANGPTL8 in metabolic syndrome as well as its association with inflammatory markers like HsCRP has not yet been well studied. As a result, we designed this population study to look at the association between HsCRP and ANGPTL8 and their relationship with MetS and its components.

## Research design and methods

### Study participants and anthropometric and physical measurements and ethics, consent and permissions

This study was performed on 1735 adult (>18 years old) South Asians (Indians and Pakistanis) and Arabs living in Kuwait as previously described [21–23]. Briefly, the samples have been continuously collected randomly from multi-ethnic subjects living in Kuwait. Subjects younger than 18 and older than 65 or suffering from any kind of infection were excluded. The non-diabetic subjects were then selected as subjects without disease and not taking any medications. Subjects with cardiovascular diseases were excluded from the study. No treatment was received before sampling. The study conformed to the principles outlined in the Declaration of Helsinki and in accordance with the approved guidelines. The study was approved by the Ethical Review Committee at Dasman Diabetes Institute (DDI). An informed written consent was obtained from all the participants before their enrolment in the study.

Physical and anthropometric measurements included body weight, height, waist circumference (WC). These were measured as described previously [21–23]; height and weight were measured, with participants wearing light indoor clothing and barefooted, using calibrated portable electronic weighing scales and portable inflexible height measuring bars. WC was measured using constant tension tape at the end of a normal exhalation, with arms relaxed at the sides, at the highest point of the iliac crest and at the mid-axillary line. BMI was calculated using the standard BMI formula: body weight (in kilograms) divided by height (in meters squared).

### Laboratory measurements

Blood samples were obtained after fasting overnight for at least 10 h and analyzed for Fasting Blood Glucose (FBG), hemoglobin A1c (HbA1c), fasting insulin, and lipid profiles that included triglyceride (TG), Total cholesterol (TC), low density lipoprotein (LDL) and high density lipoprotein (HDL). Glucose and lipid profiles were measured on the Siemens Dimension RXL chemistry analyzer (Diamond Diagnostics, Holliston, MA). HbA1c was determined using the VariantTM device (BioRad, Hercules, CA) as described earlier [21–23].

### Diabetes and MetS diagnosis and guidelines

The current recommendations and updated guidelines for the definition, diagnosis and classification of T2D, published by the International Diabetes Federation (IDF), have been used. Diabetes was defined by fasting plasma glucose ≥ 7 mmol/l, under treatment, or self-reported of previously diagnosed T2D [24]. Impaired fasting glucose (IFG) was defined by fasting blood glucose values ≥ 5.6 and <7 mmol/L. The current recommendations and updated guidelines for the definition, diagnosis and classification of MetS, published by the International Diabetes Federation (IDF), were used. MetS was defined by abdominal obesity and at least two of the following: fasting blood glucose values ≥ 5.6 mmol/L, hypertension was defined as BP ≥ 130/85 mmHg, under treatment, or a self-report of previously diagnosed hypertension [25] Hypertriglyceridemia as ≥ 1.7 mmol/L and low HDL cholesterol as <1.03 mmol/L in men and <1.29 mmol/L in women. BMI between 18.5 and 24.9 was considered normal, 25–29.9, overweight, and equal to or higher than 30, was considered obese. Cutoffs for central obesity were adopted from IDF; they were defined based on race and gender. In Arabs WC ≥ 94 cm in men and ≥80 cm in women was used, whereas for South Asians central obesity cutoffs were WC ≥ 90 cm for men and WC ≥ 80 cm for women.

### ANGPTL8 and HsCRP ELISA level

To measure metabolic markers, blood was drawn into EDTA tubes. Plasma was obtained after centrifugation, aliquoted and then stored at −80 °C. ANGPTL8 concentration was determined using ELISA (Wuhan EIAAB Science, China) as previously described [21, 22]. Optimal dilution was found to be between 1:10–1:40 and a dilution of 1:25 was used. Inter-assay coefficients of variation were 1.2 to 3.8 % while the intra-assay coefficients of variation were 6.8 to 10.2 %. HsCRP secreted level was measured using ELISA kit (Biovendor, USA) and measured according to manufacturer’s protocol as previously described [23].

### Statistical analysis

Normality tests were run to assess data distribution. Comparisons between subjects with MetS and without MetS were made by Student’s t test or Wilcoxon test for non-parametric analyses in variables with non-normal distribution. To assess the difference in categorical variables between subjects with and without MetS, a Chi Squared test was used. Spearman’s correlation coefficients were estimated to determine associations between ANGPTL8, HsCRP and anthropometric measurements and biochemical variables. Subjects were classified into tertiles based on their circulating betatrophin levels in the overall population. ANGPTL8 tertile values are T1 ≤ 689.52.4 pg/mL, T2 689.52 ≤ 1302.63 pg/mL, T3 > 1302.63 pg/mL. Tertile values of HsCRP are expressed as T1 (<1.39), T2 (1.39–4.07), and T3 (>4.07).

A multivariable logistic regression analysis was performed to estimate odds ratios (ORs) adjusted for covariates and to assess the predictive effect of betatrophin on risk for T2D. All data are reported as Mean ± standard deviation (SD) and range, unless stated otherwise. Research Electronic Data Capture (REDCap) was used for data collections and data management. All statistical assessments were two-sided and considered to be significant when *Pvalue* <0.05. All analyses were performed using SAS (version 9.2; SAS Institute, Cary, NC).

## Results

Population characteristics are outlined in Table 1. Our population was made of 1735 subjects, 724 of which were non-MetS and 1011 were MetS subjects. The average age of participants was 41.1 ± 10.6 for non-MetS subjects and 49.0 ± 10.9 for subjects with MetS. Subjects with MetS had a significantly higher BMI, waist/hip ratio, systolic and diastolic blood pressure, FBG, HBA1c, insulin, HOMA-IR, TG, TC, HDL, and LDL (p < 0.05). ANGPTL8 showed close to twofold increase in subjects with MetS relative to non-MetS subjects 1140.6 (171.9–11736.1) pg/mL vs. 710.5 (59.5–11597.2) pg/mL respectively (P value <0.0001) Table 1. HsCRP level was 1.79 (0.01–31.00) μg/mL in non-MetS subjects vs. 2.94 in MetS subjects (0.01–31.00) μg/mL (P value <0.0001) Table 1.

**Table 1:** Clinical and biochemical profile for study subjects

Variable	Control N = 724	MetS N = 1011	*P* value
Age (years)	41.1 ± 10.6	49.0 ± 10.9	<0.0001
Height (m)	1.64 ± 0.09	1.65 ± 0.09	0.0723
Weight (kg)	74.4 ± 16.3	84.9 ± 17.1	<0.0001
BMI (kg/m^2^)	27.6 ± 5.3	31.3 ± 6.02	<0.0001
Waist (cm)	91.6 ± 12.7	101.8 ± 12.4	<0.0001
Hip (cm)	102.3 ± 11.5	108.8 ± 12.3	<0.0001
Wc/hip ratio	0.90 ± 0.08	0.94 ± 0.08	<0.0001
Systolic (mmHg)	123.7 ± 17.4	137.7 ± 18.7	<0.0001
Diastolic (mmHg)	75.6 ± 10.6	82.6 ± 11.8	<0.0001
FBG (mmol/L)	5.39 ± 1.92	7.05 ± 2.9	<0.0001
Hba1c (%)	5.71 ± 1.15	6.85 ± 1.9	<0.0001
Insulin μU/mL	8.31 ± 13.6	13.56 ± 18.3	<0.0001
HOMAIR	2.13 ± 5.70	4.52 ± 2.70	<0.0001
TC (mmol/L)	5.05 ± 1.03	5.14 ± 1.12	0.0683
TG (mmol/L)	1.18 ± 0.61	1.86 ± 1.22	<0.0001
HDL (mmol/L)	1.27 ± 0.35	1.05 ± 0.27	<0.0001
LDL (mmol/L)	3.26 ± 0.93	3.28 ± 0.98	0.7189
ANGPTL8 (pg/ml)	710.5 (59.5–11597.2)	1140.6 (171.9–11736.1)	<0.0001
HsCRP (μg/mL)	1.79 (0.01–31.00)	2.94 (0.01–31.00)	<0.0001

Results are reported as Mean ± SD except for non-normally distributed ANGPTL8 and HsCRP that are presented as median (range)

**Fig. 1:**
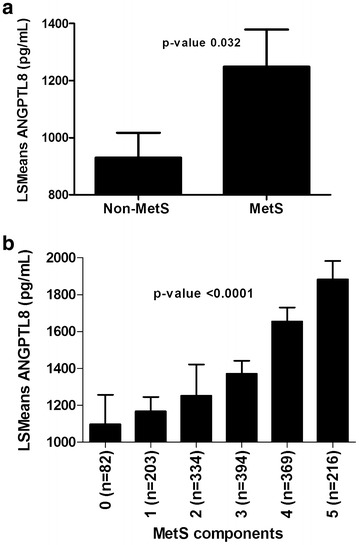
Circulating level of ANGPTL8 in subjects MetS. **a** Least square means of plasma level of ANGPTL8 in MetS vs non-MetS subjects. **b** Least square means of plasma level of ANGPTL8 according to the number of MetS components

Comparing the Least Square Means (LSMeans) between non-MetS and MetS subjects showed significant increase in the level of ANGPTL8 in the MetS subjects as shown in Fig. 1a (*P* value = 0.032). Dividing the population according to the number of MetS components showed increased ANGPTL8 level as the number of MetS components increases Fig. 1b (P value <0.0001).

Partial Spearman’s correlation adjusted for gender and ethnicity showed that ANGPTL8 was positively correlated to HsCRP level (r = 0.15, *P* < 0.001) Table 2. ANGPTL8 also showed strong positive correlation to age (r = 0.59, *P* < 0.001), BMI (r = 0.16, *P* < 0.001), waist/hip ratio (r = 0.32, *P* < 0.001), FBG (r = 0.43, *P* < 0.001), HbA1c (r = 0.38, *P* < 0.001), HOMA-IR (r = 0.33, *P* < 0.001). Similarly using partial Spearman’s analysis adjusting for gender and ethnicity HsCRP was positively associated with age, BMI, waist/hip ratio, FBG, HbA1c and HOMA-IR as shown in Table 2.

**Table 2:** Partial spearman correlation coefficients of ANGPTL8 and CRP with various parameters adjusted for ethnicity and gender

Markers	ANGPTL8		HsCRP	
	r	p value	r	p value
Age	0.59	<0.0001	0.09	0.0024
BMI	0.16	<0.0001	0.41	<0.0001
WC/HIP ratio	0.32	<0.0001	0.21	<0.0001
FBG	0.43	<0.0001	0.18	<0.0001
Hba1C	0.38	<0.0001	0.22	<0.0001
Insulin	0.19	<0.0001	0.33	<0.0001
HOMAIR	0.33	<0.0001	0.34	<0.0001
HsCRP	0.15	<0.0001	0.15	<0.0001

**Fig. 2:**
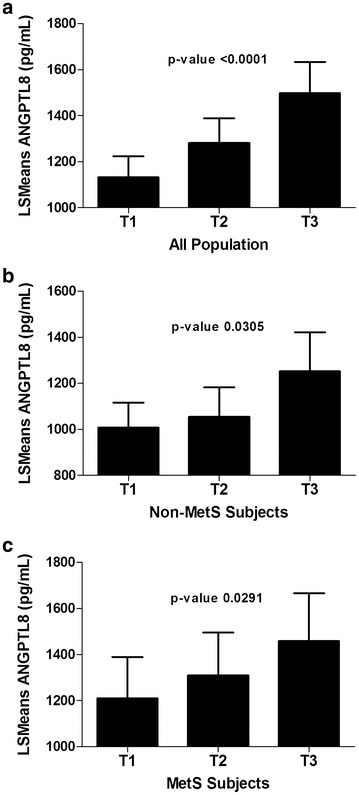
Association between ANGPTL8 and HsCRP according to MetS. **a** least square means of circulating level of ANGPTL8 according to HsCRP tertiles in all the subjects. **b** ANGPTL8 level in non-MetS subjects according to HsCRP tertiles. **c** ANGPTL8 level in MetS subjects according to HsCRP tertiles. Tertile values of HsCRP are expressed as T1 (<1.39 μg/mL), T2 (1.39–4.07 μg/mL), and T3 (>4.07 μg/mL)

In order to understand the association between ANGPTL8 and HsCRP, the population was divided according to tertiles of HsCRP. Age and ethnicity adjusted estimates of ANGPTL8 means showed significant association between levels of ANGPTL8 and HsCRP among all subgroups. Overall, subjects in the highest tertiles of HsCRP had the highest ANGPTL8 levels Fig. 2a. Dividing the population according to MetS, both subjects in the MetS and non-MetS groups showed a similar trend where subjects in the highest tertile of HsCRP had the highest level of ANGPTL8 Fig. 2b, c.

**Fig. 3:**
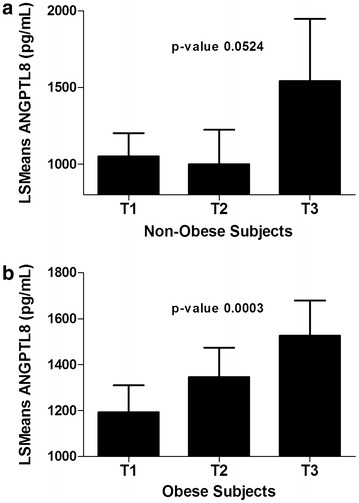
Association between ANGPTL8 and HsCRP according to obesity. **a** least square means of circulating level of ANGPTL8 according to HsCRP tertiles in non-obese subjects. **b** ANGPTL8 level in obese subjects according to HsCRP tertiles. Tertile values of HsCRP are expressed as T1 (<1.39 μg/mL), T2 (1.39–4.07 μg/mL), and T3 (>4.07 μg/mL). BMI between 18.5–29.9 was considered non-obese while BMI equal to or higher than 30, was considered obese

In order to understand the effect of obesity on the association between the ANGPTL8 and HsCRP, the population was divided according to BMI into non-obese and obese subjects. BMI between 18.5 and 29.9 was considered non-obese, while BMI equal to or higher than 30 was considered obese. Weak association was observed between ANGPTL8 and HsCRP in the non-obese (*p* value = 0.0524) Fig. 3a. On the other hand, in the obese group, a significant association was observed and subjects in the highest tertiles of HsCRP had higher levels of ANGPTL8 (*p* value = <0.0003) Fig. 3b.

Multiple logistic regression analysis of ANGPTL8 and HsCRP showed that in the unadjusted model, subjects in the highest tertile of ANGPTL8 were more likely to have MetS (OR = 4.4, 95 % CI = 3.4–5.7) Table 3. After adjustment for age, gender and ethnicity in Model-2, subjects in the highest tertile of ANGPTL8 had higher odds of having MetS (OR = 2.8, 95 % CI = 2.0–3.9). Further adjustment for HsCRP in Model-3 did not affect the association and subjects in the highest tertile still had higher odds of having MetS (OR = 2.4, 95 % CI = 1.6–3.6) (*P* trend <0.0001). Multiple logistic regression analysis for HsCRP showed that in the unadjusted model, subjects in the highest tertile of HsCRP were more likely to have MetS (OR = 2.4, 95 % CI = 1.8–3.1) Table 3. Further adjustment for age, gender and ethnicity in Model-2 and HsCRP in Model-3 did not affect the association and subjects in the highest tertile in model-3 still had higher odds of having MetS (OR = 2.3, 95 % CI = 1.6–3.1) (*P* trend <0.0001) Table 3.

**Table 3:** OR (95 % CI) by multiple logistic regression models for metabolic syndrome in relation to ANGPTL8 and HsCRP

Models	T1	T2	95 % CI	T3	95 % CI	P trend
ANGPTL8						
Model 1	1	2.3	(1.8–2.8)	4.4	(3.4–5.7)	<0.0001
Model 2	1	1.7	(1.3–2.2)	2.8	(2.0–3.9)	<0.0001
Model 3	1	1.7	(1.2–2.3)	2.4	(1.6–3.6)	<0.0001
HsCRP						
Model 1	1	2.0	(1.5–2.7)	2.4	(1.8–3.1)	<0.0001
Model 2	1	1.8	(1.3–2.5)	2.5	(1.8–3.4)	<0.0001
Model 3	1	1.8	(1.3–2.4)	2.3	(1.6–3.1)	<0.0001

*Model 1* unadjusted; *Model 2* adjusted for age, gender and ethnicity; *Model 3* adjusted for ANGPTL8 + CRP + Model 2. Tertile values of HsCRP are expressed as T1 (<1.39 μg/mL), T2 (1.39–4.07 μg/mL), and T3 (>4.07 μg/mL). Tertile values of ANGPTL8 are T1 (<689.52 pg/ml), T2 (689.52–1302.63 pg/ml), and T3 (>1302.63 pg/ml)

## Discussion

In this study we showed that ANGPTL8 circulation level was higher in subject with MetS as well as subjects with increasing number of MetS components such as insulin resistance and central obesity. ANGPTL8 showed significant association with HsCRP where both showed significant positive association with BMI, TG, LDL, HOMA-IR and FBG. Age and ethnicity adjusted estimates of ANGPTL8 were increased in concordance with HsCRP level in subjects with MetS or without as well as across different BMI groups. Finally, higher ANGPTL8 levels were associated with 2.4-fold increase in the odds of having MetS. Higher HsCRP levels on the other hand were associated with a 2.3-fold increase in the odds of having MetS. Taken together, our data clearly indicate that increased ANGPTL8 level is associated with higher level of HsCRP and incidence of MetS in our population.

ANGPTL protein family members have been shown to play a major role in obesity and metabolic diseases [26–29]. ANGPTL3 and 4 have been heavily involved in lipid metabolism through their regulation of lipoprotein lipase (LPL) activity [26]. ANGPTL8 has been recently recognized as another member of this family that plays a role in lipid metabolism and insulin signalling [14, 15, 17, 21, 22, 30]. It has been shown to regulate LPL activity through its interaction with ANGPTL3 [15]. It has also been found that an arginine (R) to tryptophan (W) R59W sequence variant in the ANGPTL8 gene has been associated with lower LDL-C and HDL-C in some ethnicities [15]. Its overexpression leads to increased TG in serum while its knockout has led to increased LPL activity and reduced fatty acid uptake by adipose tissues [30]. It has also been shown to play a role in insulin signalling and has been initially suggested to increase beta-cell proliferation [13]. However, recent studies found that ANGPTL8 had no effect on beta cell growth in mice, thus raising questions on how ANGPTL8 improves glucose tolerance [19, 20]. We and others have recently showed that ANGPTL8 level was increased in T2D [21, 31–33] and its level associated with FBG and insulin resistance in a large human cohort [21]. We also showed that it was associated with C-peptide production in non-diabetic subjects [22]. In this study we further show that ANGPTL8 is increased in MetS and is associated with HsCRP. Our current data is in line with what has been reported about the increased ANGPTL8 level in obesity and T2D [21, 31–34]. Other studies showed that ANGPTL8 was reduced in T2D [35, 36]. MetS is recognized as a cluster of metabolic risk factors including central obesity and insulin resistance [23, 37–39]. Based on its involvement in two key pathways (lipid metabolism and insulin resistance), we hypothesized that the increased ANGPTL8 level caused by insulin resistance in MetS subjects will lead to an increased release of TG into plasma. This may be done through its ability to regulate the cleavage of active ANGPTL3 that leads to inhibition of LPL enzyme activity [15]. Cleavage of ANGPTL3 causes the release of its N-terminal domain that can act as a potent inhibitor of LPL activity [15]. Even though this might be a plausible analysis, a follow up study will be necessary to establish causality highlighting one of the limitations of the current study due to its cross-sectional nature. On the other hand, one of the strengths of this study is the large sample size used as well as the population being studied, which has not been well studied yet and is being recognized as a high risk population particularly the South Asians. It important to note that the EIAAB ELISA used in this study recognizes ANGPTL8 at its N-terminus and measures the full length form. The C-terminal form of ANGPTL8 is measured using Phoenix Pharmaceuticals ELISA kit that reacts with C-terminus and measures truncated ANGPTL8 [40, 41]. We have recently showed that both forms show a similar trend in obesity and their level was reduced after exercise, however, only full length ANGPTL8 showed association with FBG [40].

HsCRP has been shown to closely associate with MetS and acts as a marker for low grade systemic inflammation [38]. Its addition to MetS definition has been suggested to increase its predictive power of CVD and T2D [9–12]. To gain better understanding on the function of ANGPTL8 in MetS we looked at its association with HsCRP as a marker of inflammation. This is because it has been suggested that the disease progression in subjects with MetS towards T2D is accompanied by increased inflammation [42]. Our data shows positive correlation between ANGPTL8 and HsCRP suggesting that the inflammatory process might be connected to the increase in ANGPTL8 in humans that could result in aggravated dyslipidemia. Similarly, ANGPTL3 and 4 have previously showed positive association with MetS [43] and ANGPTL4 showed positive association with CRP [42]. The close association between the ANGPTL8 and HsCRP shows that ANGPTL8 may be used in the future as a predictor of MetS and possibly CVD in combination with HsCRP.

In conclusion, our data shows that circulating ANGPTL8 level is increased in subjects with MetS or some of its components and this increase coincide with increase in the HsCRP level where both proteins can act as independent predictors of MetS. This data highlights the potential role of ANGPTL8 in MetS and the potential usage of this molecule as a predictive biomarker for MetS and CVD in the future.

## References

[1] Mottillo S, Filion KB, Genest J, Joseph L, Pilote L, Poirier P, Rinfret S, Schiffrin EL, Eisenberg MJ (2010). The metabolic syndrome and cardiovascular risk a systematic review and meta-analysis. J Am Coll Cardiol.

[2] Ballantyne CM, Hoogeveen RC, Bang H, Coresh J, Folsom AR, Chambless LE, Myerson M, Wu KK, Sharrett AR, Boerwinkle E (2005). Lipoprotein-associated phospholipase A2, high-sensitivity C-reactive protein, and risk for incident ischemic stroke in middle-aged men and women in the Atherosclerosis Risk in Communities (ARIC) study. Arch Intern Med.

[3] Downs JR, Clearfield M, Weis S, Whitney E, Shapiro DR, Beere PA, Langendorfer A, Stein EA, Kruyer W, Gotto AM (1998). Primary prevention of acute coronary events with lovastatin in men and women with average cholesterol levels: results of AFCAPS/TexCAPS. Air Force/Texas Coronary Atherosclerosis Prevention Study. JAMA.

[4] Rezaianzadeh A, Namayandeh SM, Sadr SM (2012). National Cholesterol Education Program Adult Treatment Panel III Versus International Diabetic Federation Definition of Metabolic Syndrome, Which One is Associated with Diabetes Mellitus and Coronary Artery Disease?. Int J Prev Med.

[5] Parikh R, Mohan V, Joshi S (2012). Should waist circumference be replaced by index of central obesity (ICO) in definition of metabolic syndrome?. Diabetes Metab Res Rev.

[6] Zimmet P, Alberti G (2008). The metabolic syndrome: progress towards one definition for an epidemic of our time. Nat Clin Pract Endocrinol Metab.

[7] Miida T, Seino U, Miyazaki O, Hanyu O, Hirayama S, Saito T, Ishikawa Y, Akamatsu S, Nakano T, Nakajima K, Okazaki M, Okada M (2008). Probucol markedly reduces HDL phospholipids and elevated prebeta1-HDL without delayed conversion into alpha-migrating HDL: putative role of angiopoietin-like protein 3 in probucol-induced HDL remodeling. Atherosclerosis.

[8] Yudkin JS, Juhan-Vague I, Hawe E, Humphries SE, di Minno G, Margaglione M, Tremoli E, Kooistra T, Morange PE, Lundman P, Mohamed-Ali V, Hamsten A (2004). Low-grade inflammation may play a role in the etiology of the metabolic syndrome in patients with coronary heart disease: the HIFMECH study. Metabolism.

[9] Povel CM, Beulens JW, van der Schouw YT, Dolle ME, Spijkerman AM, Verschuren WM, Feskens EJ, Boer JM (2013). Metabolic syndrome model definitions predicting type 2 diabetes and cardiovascular disease. Diabetes Care.

[10] Rodriguez-Leal GA, Moran S, Gallardo I, Milke P, Guevara-Gonzalez L (2006). Assessment of high sensitivity C-reactive protein (HS-CRP) as a marker of liver inflammation in patients with metabolic syndrome. Rev Gastroenterol Mex.

[11] Rasouli M, Kiasari AM (2006). Interactions of serum hsCRP with apoB, apoB/AI ratio and some components of metabolic syndrome amplify the predictive values for coronary artery disease. Clin Biochem.

[12] Devaraj S, Swarbrick MM, Singh U, Adams-Huet B, Havel PJ, Jialal I (2008). CRP and adiponectin and its oligomers in the metabolic syndrome: evaluation of new laboratory-based biomarkers. Am J Clin Pathol.

[13] Yi P, Park JS, Melton DA (2013). Betatrophin: a hormone that controls pancreatic beta cell proliferation. Cell.

[14] Ren G, Kim JY, Smas CM (2012). Identification of RIFL, a novel adipocyte-enriched insulin target gene with a role in lipid metabolism. Am J Physiol Endocrinol Metab.

[15] Quagliarini F, Wang Y, Kozlitina J, Grishin NV, Hyde R, Boerwinkle E, Valenzuela DM, Murphy AJ, Cohen JC, Hobbs HH (2012). Atypical angiopoietin-like protein that regulates ANGPTL3. Proc Natl Acad Sci USA.

[16] Zhang R, Abou-Samra AB (2013). Emerging roles of Lipasin as a critical lipid regulator. Biochem Biophys Res Commun.

[17] Zhang R (2012). Lipasin, a novel nutritionally-regulated liver-enriched factor that regulates serum triglyceride levels. Biochem Biophys Res Commun.

[18] Abubaker J, Tiss A, Abu-Farha M, Al-Ghimlas F, Al-Khairi I, Baturcam E, Cherian P, Elkum N, Hammad M, John J, Kavalakatt S, Khadir A, Warsame S, Dermime S, Behbehani K, Dehbi M (2013). DNAJB3/HSP-40 cochaperone is downregulated in obese humans and is restored by physical exercise. PLoS One.

[19] Gusarova V, Alexa CA, Na E, Stevis PE, Xin Y, Bonner-Weir S, Cohen JC, Hobbs HH, Murphy AJ, Yancopoulos GD, Gromada J (2014). ANGPTL8/betatrophin does not control pancreatic beta cell expansion. Cell.

[20] Yi P, Park JS, Melton DA (2014). Perspectives on the activities of ANGPTL8/betatrophin. Cell.

[21] Abu-Farha M, Abubaker J, Al-Khairi I, Cherian P, Noronha F, Hu FB, Behbehani K, Elkum N (2015). Higher plasma betatrophin/ANGPTL8 level in Type 2 Diabetes subjects does not correlate with blood glucose or insulin resistance. Sci Rep.

[22] Abu-Farha M, Abubaker J, Noronha F, Al-Khairi I, Cherian P, Alarouj M, Bennakhi A, Elkum N (2015). Lack of associations between betatrophin/ANGPTL8 level and C-peptide in type 2 diabetic subjects. Cardiovasc Diabetol.

[23] Abu-Farha M, Behbehani K, Elkum N (2014). Comprehensive analysis of circulating adipokines and hsCRP association with cardiovascular disease risk factors and metabolic syndrome in Arabs. Cardiovasc Diabetol.

[24] Diagnosis and classification of diabetes mellitus (2008). Diabetes Care.

[25] Chobanian AV, Bakris GL, Black HR, Cushman WC, Green LA, Izzo JL, Jones DW, Materson BJ, Oparil S, Wright JT, Roccella EJ (2003). The Seventh Report of the Joint National Committee on Prevention, Detection, Evaluation, and Treatment of High Blood Pressure: the JNC 7 report. JAMA.

[26] Li Y, Teng C (2014). Angiopoietin-like proteins 3, 4 and 8: regulating lipid metabolism and providing new hope for metabolic syndrome. J Drug Target.

[27] Santulli G (2014). Angiopoietin-like proteins: a comprehensive look. Front Endocrinol (Lausanne).

[28] Kadomatsu T, Tabata M, Oike Y (2011). Angiopoietin-like proteins: emerging targets for treatment of obesity and related metabolic diseases. FEBS J.

[29] Oike Y, Akao M, Kubota Y, Suda T (2005). Angiopoietin-like proteins: potential new targets for metabolic syndrome therapy. Trends Mol Med.

[30] Zhang R, Abou-Samra AB (2014). A dual role of lipasin (betatrophin) in lipid metabolism and glucose homeostasis: consensus and controversy. Cardiovasc Diabetol.

[31] Espes D, Martinell M, Carlsson PO (2014). Increased circulating betatrophin concentrations in patients with type 2 diabetes. Int J Endocrinol.

[32] Fu Z, Berhane F, Fite A, Seyoum B, Abou-Samra AB, Zhang R (2014). Elevated circulating lipasin/betatrophin in human type 2 diabetes and obesity. Sci Rep.

[33] Hu H, Sun W, Yu S, Hong X, Qian W, Tang B, Wang D, Yang L, Wang J, Mao C, Zhou L (2014). Increased circulating levels of betatrophin in newly diagnosed type 2 diabetic patients. Diabetes Care.

[34] Gao T, Jin K, Chen P, Jin H, Yang L, Xie X, Yang M, Hu C, Yu X (2015). Circulating Betatrophin Correlates with Triglycerides and Postprandial Glucose among Different Glucose Tolerance Statuses–a Case-Control Study. PLoS One.

[35] Gokulakrishnan K, Manokaran K, Pandey GK, Amutha A, Ranjani H, Anjana RM, Mohan V (2015). Relationship of betatrophin with youth onset type 2 diabetes among Asian Indians. Diabetes Res Clin Pract.

[36] Gómez-Ambrosi J, Pascual E, Catalán V, Rodríguez A, Ramírez B, Silva C, Gil MJ, Salvador J, Frühbeck G (2014). Circulating betatrophin concentrations are decreased in human obesity and type 2 diabetes. J Clin Endocrinol Metab.

[37] Alberti KG, Zimmet P, Shaw J (2005). The metabolic syndrome–a new worldwide definition. Lancet.

[38] Devaraj S, Valleggi S, Siegel D, Jialal I (2010). Role of C-reactive protein in contributing to increased cardiovascular risk in metabolic syndrome. Curr Atheroscler Rep.

[39] Eckel RH, Grundy SM, Zimmet PZ (2005). The metabolic syndrome. Lancet.

[40] Abu-Farha M, Sriraman D, Cherian P, AlKhairi I, Elkum N, Behbehani K, Abubaker J (2016). Circulating ANGPTL8/Betatrophin Is Increased in Obesity and Reduced after Exercise Training. PLoS One.

[41] Fu Z, Abou-Samra AB, Zhang R (2014). An explanation for recent discrepancies in levels of human circulating betatrophin. Diabetologia.

[42] Tjeerdema N, Georgiadi A, Jonker JT, van Glabbeek M (2014). Alizadeh Dehnavi R, Tamsma JT, Smit JW, Kersten S, Rensen PC: Inflammation increases plasma angiopoietin-like protein 4 in patients with the metabolic syndrome and type 2 diabetes. BMJ Open Diabetes Res Care.

[43] Mehta N, Qamar A, Qu L, Qasim AN, Mehta NN, Reilly MP, Rader DJ (2014). Differential association of plasma angiopoietin-like proteins 3 and 4 with lipid and metabolic traits. Arterioscler Thromb Vasc Biol.

